# Two datasets of defect reports labeled by a crowd of annotators of unknown reliability

**DOI:** 10.1016/j.dib.2018.03.109

**Published:** 2018-03-28

**Authors:** Jerónimo Hernández-González, Daniel Rodriguez, Iñaki Inza, Rachel Harrison, Jose A. Lozano

**Affiliations:** a Department of Computer Science and Artificial Intelligence, University of the Basque Country UPV/EHU, Donostia, Spain; b Basque Center for Applied Mathematics BCAM, Bilbao, Spain; c Department of Computer Science, University of Alcala, Madrid, Spain; d Department of Computing, Oxford Brookes University, Oxford, UK

## Abstract

Classifying software defects according to any defined taxonomy is not straightforward. In order to be used for automatizing the classification of software defects, two sets of defect reports were collected from public issue tracking systems from two different real domains. Due to the lack of a domain expert, the collected defects were categorized by a set of annotators of unknown reliability according to their impact from IBM's orthogonal defect classification taxonomy. Both datasets are prepared to solve the defect classification problem by means of techniques of the learning from crowds paradigm (Hernández-González et al. [1]).

Two versions of both datasets are publicly shared. In the first version, the raw data is given: the text description of defects together with the category assigned by each annotator. In the second version, the text of each defect has been transformed to a descriptive vector using text-mining techniques.

**Specifications Table**

**Table:** 

Subject area	*Computer Science*
More specific subject area	*Software Engineering; Machine Learning*
Type of data	*Text fields and multiple annotations of a discrete class variable (defect impact* of IBM's orthogonal defect classification, *ODC*[2]*).*
How data was acquired	*Gathered from public issue tracking systems for the defect descriptions. Manual annotation of each defect by different labelers.*
Data format	*Both raw and text-processed datasets.*
Experimental factors	*In the second version of the datasets, standard natural language processing techniques were used to extract a relevant set of variables from the text fields and transform the original database into a dataset which can be handled by machine learning techniques.*
Experimental features	*A description of the datasets and the agreement between the labels of the different annotators is provided.*
Data source location	http://compendium.open.ac.uk/bugzilla/
	http://bugzilla.mozilla.org/
Data accessibility	*All the data is published together with this article.*
Related research article	*The data in this paper was used in**[1]*.

**Value of the Data**

• A large set of software defect reports is collected (and processed) from public repositories and adapted for the task of defect classification.
• Five labelers for each dataset give their annotations by means of the most convenient defect impact from the ODC taxonomy [2], according to their subjective point of view.
• As no ground truth is available, the evaluation of classification models learnt from this type of data is a challenge that has not been solved to date.
• The processing and extraction of meaningful information from the text fields that may boost the performance of the learning algorithms may be improved.

## 1 Data

In software engineering, having available the classification of the reported defects is useful, although this task is complex and time consuming. The datasets presented in this paper were prepared to learn to automatize the classification of software defects by means of the paradigm of (machine) learning from crowds. Thus, multiple annotators were asked to provide the most convenient category (label) for each defect report according to their subjective point of view. Note, therefore, that the labels provided by the annotators may be wrong. To enhance generalization, two datasets were prepared.

The first dataset is composed of reports collected from the issue tracking system (ITS) of the Compendium project, a software tool for mapping information, ideas and arguments. An ITS is typically used by software projects for reporting and tracking defects as well as proposing new functionalities. An ITS organizes the information through tickets, which maintain data such as an identifier, summary, description, opening/closing/modification dates, etc. The ITS used by the Compendium project is implemented in Bugzilla (https://www.bugzilla.org/) and collects support issues, feature requests and bug reports from the Compendium community. The collected dataset comprises 962 examples, all the entries available in the ITS in August 2014 (with the exception of some obvious spam). Only informative fields are taken into account: severity, summary and description. Severity is a 3-value variable (Bug, Support or Feature), and both summary and description are text fields. Five annotators with experience in computer science were asked to annotate the examples according to the 13-category ODC standard [2]. See Table 5 for a description of the different possible categories. Only 9 out of the original 13 categories were used by the labelers in the dataset: Installability, Integrity/Security, Migration, Reliability, Performance, Documentation, Requirements, Usability and Capability. Table 1 shows the number of examples that each annotator assigned to each class label. Although the number of examples assigned by the different annotators is almost the same for some class labels (Installability, Performance and, to a lesser extent, Integrity/Security and Documentation), there exists high variability in the majority of class labels. Moreover, a similar number of annotations does not imply consensus. Table 2 shows the assignment of examples to labels based on the consensus among annotators: each cell shows the number of examples assigned to a class label by a certain number of annotators. The last row shows the number of examples in which the consensus label is supported by a majority of annotators (three or more). This row provides an insight into the lack of homogeneity in the distribution of class labels. It can be observed that Integrity/Security, Migration, Performance and Documentation are minority classes.

**Table 1:** In the Compendium dataset, the number of examples assigned by each annotator to the different ODC defect impacts (class labels).

Defect impact	Annotator #1	Annotator #2	Annotator #3	Annotator #4	Annotator #5
Installability	92	82	86	87	87
Integrity/Security	6	14	10	12	9
Migration	91	20	89	21	25
Reliability	119	4	117	86	97
Performance	14	15	13	14	14
Documentation	29	36	19	28	25
Requirements	192	236	139	239	242
Usability	392	267	473	279	353
Capability	27	288	16	196	110

**Table 2:** In the Compendium dataset, the number of examples in which a subset of annotators agree on their given class label. The last column shows the number of examples in which the majority of annotators agree on the assigned label.

Defect impact	Number of annotators which agree on the impact of the defect				
	2	3	4	5	[3,5]
Installability	6	6	20	59	85
Integrity/Security	0	3	2	5	10
Migration	27	11	11	2	24
Reliability	26	32	39	1	72
Performance	2	1	3	9	13
Documentation	4	4	12	9	25
Requirements	61	66	100	37	203
Usability	40	69	129	96	294
Capability	10	48	10	2	60

The second dataset has been collected from the Mozilla project (http://www.mozilla.org/), a popular open-source application which started back in the late 90 s with the Netscape browser. Nowadays, it is a suite of tools that includes the Firefox browser and the Thunderbird e-mail client. This second dataset, which contains 675 defects, was also labeled by 5 annotators. In this case, 10 different defect impacts appeared among the labels provided by the annotators: Installability, Integrity/Security, Maintenance, Migration, Reliability, Performance, Documentation, Requirements, Usability and Capability (the same 9 impacts annotated in the Compendium dataset and Maintenance). Note that there are 3 categories which were not used in any of the datasets: Serviceability, Standards and Accessibility.

Table 3 shows the number of examples that each annotator assigned to each class label for both datasets. In some cases, such as the Maintenance reports, the variability is extreme. In general, the agreement among annotators is not as high as in the Compendium dataset, as can be observed in Table 4. This new table shows the assignment of examples to labels based on the consensus among annotators: each cell shows the number of examples assigned to a class label by a certain number of annotators. The last row shows the number of examples in which the consensus label is supported by a majority of annotators (three or more). The lack of homogeneity in the distribution of class labels is obvious in this Mozilla dataset. It can be observed that Integrity/Security, Migration and Performance are again minority classes.

**Table 3:** In the Mozilla dataset, the number of examples assigned by each annotator to the different ODC defect impacts (class labels).

Defect impact	Annotator #1	Annotator #2	Annotator #3	Annotator #4	Annotator #5
Installability	158	108	73	115	158
Integrity/Security	15	4	16	6	11
Maintenance	2	140	184	36	62
Migration	1	34	22	1	30
Reliability	130	159	201	375	130
Performance	11	9	13	4	14
Documentation	12	41	47	20	40
Requirements	98	42	51	77	96
Usability	88	62	6	31	44
Capability	160	76	62	10	90

**Table 4:** In the Mozilla dataset, the number of examples in which a subset of annotators agree on their given class label. The last column shows the number of examples in which the majority of annotators agree on the assigned label.

Defect impact	Number of annotators which agree on the impact of the defect				
	2	3	4	5	[3,5]
Installability	64	32	16	52	100
Integrity/Security	3	3	1	2	6
Maintenance	45	18	4	0	22
Migration	14	3	1	0	4
Reliability	64	62	40	52	154
Performance	0	1	4	2	7
Documentation	6	9	11	9	29
Requirements	29	21	15	1	37
Usability	21	13	8	0	21
Capability	15	13	1	0	14

**Table 5:** ODC defect impact classification [2].

Defect impact	Description
Installability	The ability of the customer to prepare and place the software in position for use (not include Usability).
Integrity/Security	The protection of systems, programs, and data from inadvertent or malicious destruction, alteration, or disclosure.
Maintenance	The ease of applying preventive or corrective fixes to the software.
Serviceability	The ability to diagnose failures easily and quickly, with minimal impact to the customer.
Migration	The ease of upgrading to a current release, particularly in terms of the impact on existing customer data and operations.
Reliability	The ability of the software to consistently perform its intended function without unplanned interruption. Severe interruptions, such as ABEND and WAIT would always be considered reliability.
Performance	The speed of the software as perceived by the customer and the customer's end users, in terms of their ability to perform their tasks.
Documentation	The degree to which the publication aids provided for understanding the structure and intended uses of the software are correct and complete.
Requirements	A customer expectation, with regard to capability, which was not known, understood, or prioritized as a requirement for the current release.
Usability	The degree to which the software and publication aids enable the product to be easily understood and conveniently employed by its end user.
Standards	The degree to which the software complies with established pertinent standards.
Accessibility	Ensuring that successful access to information and use of information technology is provided to people who have disabilities.
Capability	The ability of the software to perform its intended functions, and satisfy known requirements, where the customer is not impacted in any of the previous categories.

## 2 Experimental design, materials, and methods

A sufficiently large set of defects was collected from the corresponding ITS. Only informative fields were collected: summary, description and, in the case of the Compendium dataset, severity. Once the reports were collected, some obvious spam reports were removed. The remaining defect reports (up to 962 and 675 defects, respectively) were separately presented to the different annotators. Each of them was asked to label all the reports of the given dataset. Note that no annotator had access to the labels provided by any other labeler. Moreover, annotators of both datasets are not the same.

At the same time, the defects are originally described by the text reported by the user using two text fields: summary and description. Standard natural language processing techniques have been used to extract a relevant set of variables from the text fields and transform the original database into a dataset which can be handled by ML techniques. Specifically, the popular StringToWordVector filter implemented in Weka [3] was used. Stop-words were removed based on Rainbow [4], text was converted to lowercase; the iterated version of the Lovins stemmer [5] was applied as well as an alphabetic tokenizer, where tokens are formed only using contiguous alphabetic sequences. For each word a numeric variable is created which, for each defect, takes as value the Term Frequency-Inverse Document Frequency (TF-IDF) ratio. Finally, the lack of a ground truth prevented us from using feature selection techniques to filter out uninformative descriptors. The maximum number of selected attributes was just set to 100.
